# Occlusogram-Guided Interdisciplinary Planning in Implant Dentistry and Orthodontics for Oral Rehabilitation

**DOI:** 10.7759/cureus.106882

**Published:** 2026-04-12

**Authors:** Gustavo H Gameiro, Annicele s Andrade

**Affiliations:** 1 Physiology, Universidade Federal do Rio Grande do Sul, Porto Alegre, BRA; 2 Oral Health, Grupo Hospitalar Conceição (GHC), Porto Alegre, BRA

**Keywords:** implant dentistry, interdisciplinary treatment planning, occlusogram, oral rehabilitation, orthodontics

## Abstract

A 65-year-old female patient with stable periodontal status, anterior crossbite, mandibular spacing, and need for posterior implant rehabilitation was treated using an occlusogram-guided interdisciplinary approach. The increasing demand for complex oral rehabilitation has reinforced the need for effective interdisciplinary planning between orthodontics and implant dentistry. In such cases, dental implants may serve not only as prosthetic units but also as sources of absolute anchorage during orthodontic treatment, requiring precise preorthodontic positioning. This case report describes the use of an occlusogram-guided approach to optimize implant placement and coordinate orthodontic mechanics. Digital occlusograms were constructed using GeoGebra (International GeoGebra Institute, Linz) from occlusal photographs of dental casts calibrated to a 1:1 scale, allowing simulation of planned tooth movements in the transverse and sagittal planes and visualization of the final occlusal relationships. This approach enabled the determination of implant position based on the predicted posttreatment arch form, the required interproximal spacing, and the anticipated occlusal relationships. A mandibular implant was placed prior to orthodontic treatment, and after an eight-week osseointegration period, was successfully used as anchorage for posterior mesialization through a preactivated lingual arch. Treatment resulted in complete mandibular space closure (8 mm), correction of maxillary midline deviation (2 mm), establishment of bilateral Class I canine relationships, and functional occlusal integration. The implant was subsequently maintained for definitive prosthetic rehabilitation. The occlusogram also served as an effective communication tool among clinicians and facilitated the informed consent discussion by providing a visual representation of planned treatment changes. This report illustrates the clinical application of occlusogram-guided interdisciplinary planning and demonstrates its potential value in facilitating precise implant positioning and coordinated treatment sequencing in complex orthodontic-implant cases.

## Introduction

Adult patients requiring both orthodontic treatment and implant-supported rehabilitation frequently present with complex combinations of tooth loss, malocclusion, occlusal disturbances, and reduced anchorage availability. Successful management of these cases depends on coordinated interdisciplinary planning that integrates diagnostic, orthodontic, and surgical considerations from the outset [1-5]. When substantial tooth movement is required, such as posterior mesialization to close edentulous spaces, the dual challenge of providing adequate orthodontic anchorage while ensuring correct implant positioning for future prosthetic rehabilitation becomes particularly critical.

Traditionally, implants were placed only after orthodontic treatment was completed, often prolonging total treatment time and limiting the scope of achievable orthodontic movements when posterior segments were edentulous [6]. The introduction of osseointegrated implants as orthodontic anchorage provided new opportunities for efficient tooth movement in such patients, as adequately osseointegrated implants resist orthodontic forces while allowing natural teeth to be moved around them when appropriate healing time and biologically acceptable force levels are observed [7-10]. Studies have confirmed that controlled orthodontic loading does not compromise osseointegration when biologically acceptable force levels are used [9]. Typical protocols involve healing periods of 8-12 weeks before orthodontic loading, or immediate/early loading when adequate primary stability is achieved (insertion torque >35 Ncm), with orthodontic forces generally ranging from 200-300 g for molar movement [4,9].

However, for implants to serve both as anchorage and future prosthetic abutments, their placement must be determined with reference to the final occlusal scheme, not the patient's initial malocclusion. Implant placement based solely on existing tooth positions risks misalignment after orthodontic treatment, potentially resulting in inadequate interproximal space, suboptimal emergence profiles, unfavorable root proximity, or compromised prosthetic outcomes [11,12].

To address this challenge, the occlusogram has been proposed as an objective diagnostic tool that enables clinicians to simulate planned tooth movements and visualize the final occlusion. Operationally, the occlusogram uses calibrated occlusal photographs and mesiodistal tooth measurements to generate a graphical representation of both dental arches, allowing simulation of planned tooth movements and prediction of final arch form, thereby guiding implant positioning compatible with posttreatment occlusal relationships. Originally described by Marcotte [5] and later expanded into digital versions by Fiorelli and Melsen [13], the occlusogram visualizes planned tooth movements, space distribution, arch form changes, and anticipated occlusal relationships, helping determine ideal implant positioning before orthodontic mechanics begin.

Contemporary interdisciplinary treatment planning increasingly employs advanced three-dimensional (3D) digital workflows, including cone beam computed tomography (CBCT)-guided implant surgery, intraoral scanning with virtual orthodontic setups (e.g., ClinCheck, SureSmile), and computer-aided design/computer-aided manufacturing prosthetically driven surgical guides [1,2,12]. These technologies provide comprehensive 3D visualization and have become the standard in well-resourced clinical settings. However, such systems require substantial investment in hardware, software, and training, potentially limiting accessibility in many clinical environments. The two-dimensional (2D) digital occlusogram represents an accessible alternative that can be constructed using basic imaging equipment and freely available software (e.g., GeoGebra, International GeoGebra Institute, Linz), while still providing adequate visualization of arch form changes and occlusal relationships needed for integrated implant-orthodontic planning. Thus, the occlusogram may serve as a practical planning tool when advanced 3D technology is unavailable or when clinical circumstances favor a simpler workflow.

Despite its practicality and accessibility, occlusogram-guided implant planning appears underreported in contemporary literature, with limited published case reports or clinical series describing its systematic application in integrated implant-orthodontic treatment. The present report describes the interdisciplinary management of an adult patient requiring mandibular implant placement before orthodontic movement. Specifically, this case illustrates how a 2D digital occlusogram, constructed using accessible equipment and freely available software, was used to establish the correct implant position and coordinate orthodontic and surgical sequencing. The case demonstrates successful achievement of planned outcomes, including 8 mm mandibular space closure, midline correction, and functional occlusal integration with the implant positioned as predicted for definitive prosthetic rehabilitation.

## Case presentation

Patient overview

A 65-year-old female patient was referred for orthodontic evaluation after periodontal stabilization. The patient had been diagnosed with Stage II, Grade B periodontitis (localized moderate periodontitis per 2018 AAP/EFP classification) and completed nonsurgical periodontal therapy consisting of full-mouth scaling and root planing, followed by maintenance therapy at three-month intervals for 12 months prior to orthodontic referral. At the time of orthodontic evaluation, periodontal examination revealed probing depths ≤4 mm throughout the dentition with no sites exceeding 5 mm, bleeding on probing <15%, and adequate zones of attached gingiva. Teeth adjacent to the planned implant site (#44, #45) and the primary tooth to be orthodontically moved (#36) demonstrated probing depths of 2-3 mm with no mobility. Radiographic evaluation showed horizontal bone loss with alveolar crest levels approximately 3-4 mm apical to the cementoenamel junction in posterior regions, consistent with stabilized moderate periodontitis. The periodontal prognosis was considered favorable for combined orthodontic and implant treatment.

**Figure 1:**
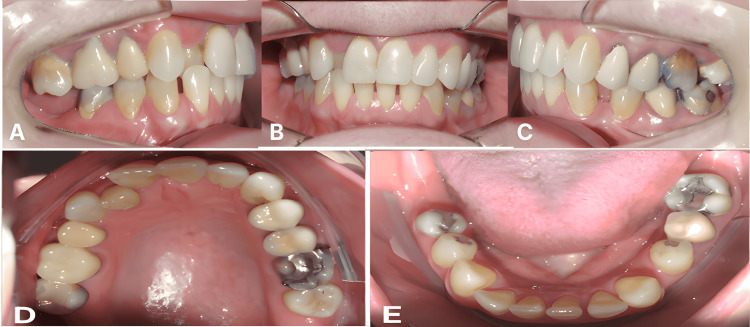
Initial intraoral clinical presentation showing baseline malocclusion (A) Right buccal view showing Class II canine relationship, and moderate maxillary crowding. (B) Frontal view showing anterior crossbite with tooth #12 (maxillary right lateral incisor) positioned palatally to tooth #42 (mandibular left lateral incisor), and 2 mm maxillary midline deviation to the right (coincident with mandibular midline). (C) Left buccal view showing Class II molar relationship between teeth #17 and #27. (D) Maxillary occlusal view showing moderate crowding, full-coverage crowns on teeth #24 and #25, and absence of tooth #15. (E) Mandibular occlusal view showing generalized spacing concentrated on the right side, absence of teeth #37, #46, and #47, and amalgam restorations on posterior teeth

She reported concerns with a crossbite affecting the maxillary right lateral incisor, spacing in the mandibular arch, and the desire for implant-supported rehabilitation in the posterior mandible. Functional assessment revealed no temporomandibular joint signs or symptoms (no pain, clicking, crepitus, or limitation of opening). A slight anterior mandibular shift of 1-2 mm was observed from centric relation to maximum intercuspation (centric occlusion), likely associated with the anterior crossbite. Clinical examination revealed moderate crowding in the maxillary arch, including an anterior crossbite in which tooth #12 (maxillary right lateral incisor) occluded palatally to tooth #42 (mandibular right lateral incisor), a 2 mm deviation of the maxillary midline to the right, and generalized spacing in the mandibular arch concentrated on the right side, including absence of teeth #46 and #47 (mandibular right first and second molars). Tooth #17 (maxillary right second molar) showed supraeruption of approximately 2-3 mm into the space created by the long-standing absence of mandibular antagonists (Figure 1).

Teeth #46 and #47 had been extracted approximately five years prior to presentation due to advanced caries and vertical root fracture, respectively. Clinical and radiographic examination of the edentulous ridge revealed adequate alveolar bone height and width for implant placement, with healthy keratinized mucosa and minimal resorptive changes. The mesiodistal space in the edentulous area measured approximately 20 mm, sufficient to accommodate a single implant at position #46 while permitting planned mesial movement of the left posterior segment to close additional mandibular spacing.

**Figure 2:**
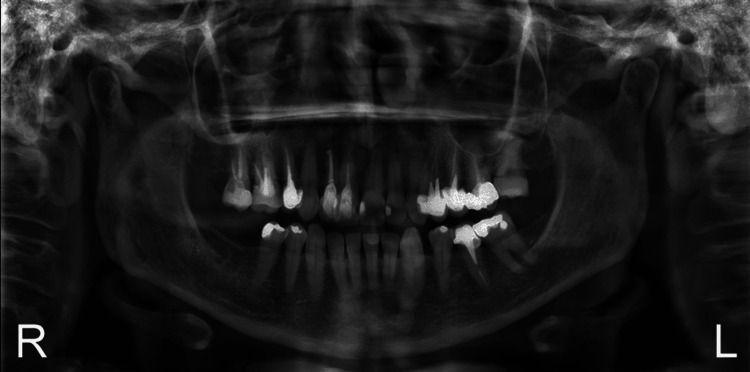
Initial panoramic radiograph demonstrating absence of teeth #46 and #47, adequate bone for implant placement, and no periapical pathology

**Figure 3:**
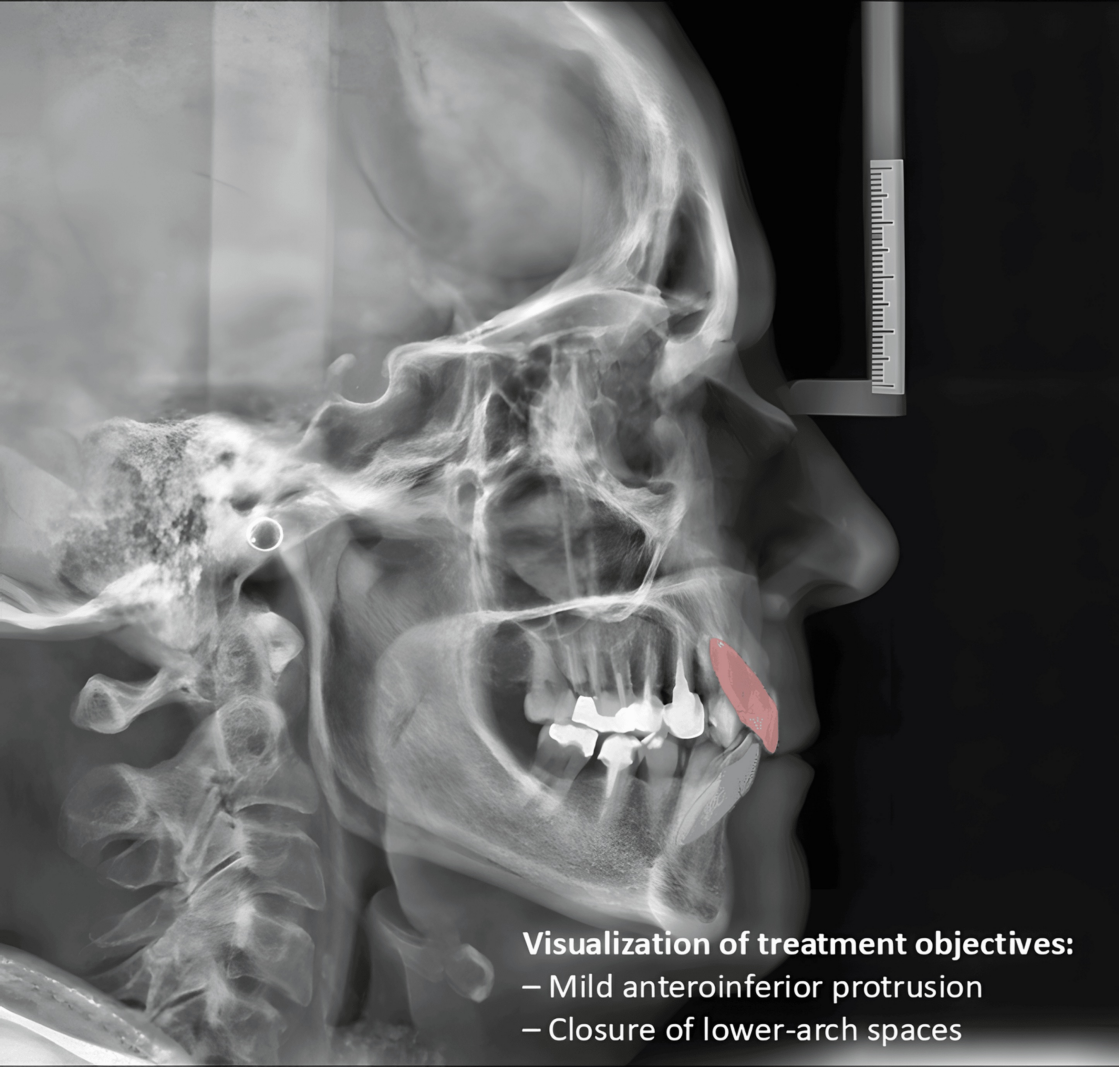
Initial lateral cephalometric radiograph with visual treatment objective superimposed, showing planned anteroposterior and vertical tooth movements

A panoramic radiograph confirmed adequate bone height in the edentulous mandibular region (Figure 2), while lateral cephalometric analysis demonstrated a skeletal Class I relationship and mild bimaxillary retrusion. Based on the patient’s facial profile, mild advancement of the incisors was considered acceptable within the treatment plan (Figure 3).

Interdisciplinary planning and occlusogram analysis

Two maxillary treatment options were initially discussed: extraction of tooth #25 or progressive interproximal reduction (IPR). Given the full-coverage crowns on teeth #24-#27, IPR was selected and performed with approximately 0.5 mm reduction per surface from the mesial of #27 to the distal of #23 (eight surfaces), generating approximately 4 mm of space to resolve crowding and reposition tooth #12 out of crossbite.

**Figure 4:**
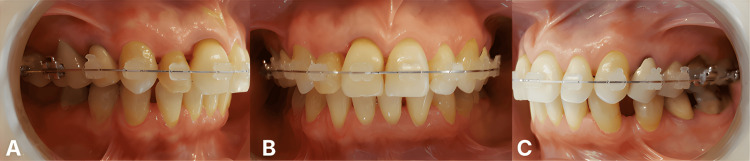
Mid-treatment (maxillary completion) Intraoral presentation after 12 months of maxillary orthodontic treatment. (A) Right buccal view showing maxillary alignment with fixed appliances. (B) Frontal view showing correction of anterior crossbite, and maxillary midline centered with facial midline (correcting initial 2 mm deviation). (C) Left buccal view showing completed maxillary alignment and space management through progressive interproximal reduction. Patient is ready for the mandibular implant placement phase

Once maxillary alignment and crossbite correction were achieved (Figure 4), digital occlusograms were constructed to evaluate mandibular tooth movement requirements and determine the appropriate implant sites. The occlusogram methodology involved 1) standardized occlusal photographs of maxillary and mandibular dental casts taken perpendicular to the occlusal plane; 2) calibration to the 1:1 scale using a known reference dimension (mesiodistal width of tooth #46 region measured directly on the cast); 3) digital tracing of individual tooth outlines using GeoGebra software (freely available open-source geometry application); 4) superimposition of maxillary and mandibular arch tracings to simulate occlusal relationships; and (5) simulation of planned tooth movements by digitally repositioning individual tooth outlines to predict the final arch form and occlusal scheme.

The occlusogram analysis revealed that approximately 8 mm of mandibular space closure through mesial movement of the left posterior segment (teeth #36, #35, #34, #33) would be required. Position #46 (mandibular right first molar) was selected for primary implant placement because 1) this site represents the location of greatest masticatory force, essential for functional restoration; 2) the implant could provide absolute anchorage for the planned 8 mm mesialization; and 3) the occlusogram confirmed that positioning the implant according to the predicted final arch form would ensure proper occlusal integration after orthodontic completion.

**Figure 5:**
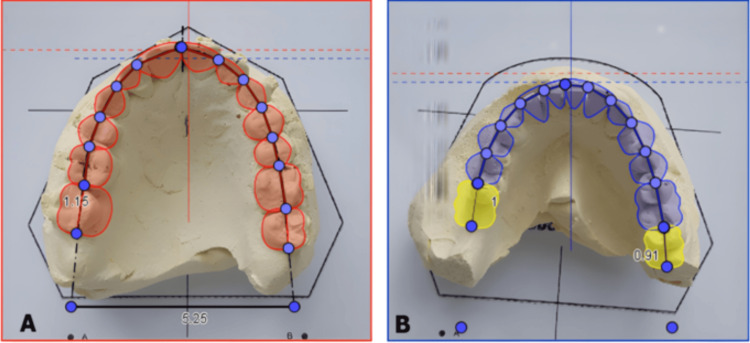
Digital occlusogram Digital occlusogram showing maxillary (A; red) and mandibular (B; blue) arch forms with planned tooth movements. Yellow tracings indicate ideal implant positions at #46 and future site #37, determined by analysis of the final predicted occlusion Image credit: The image was created using GeoGebra Classic 6 (International GeoGebra Institute, Linz, Austria)

Additionally, the occlusogram identified the need for future implant placement at site #37: complete space closure would leave ~10 mm of edentulous space distal to #36, where bilateral posterior support could not be achieved without prosthetic rehabilitation (Figure 5).

**Figure 6:**
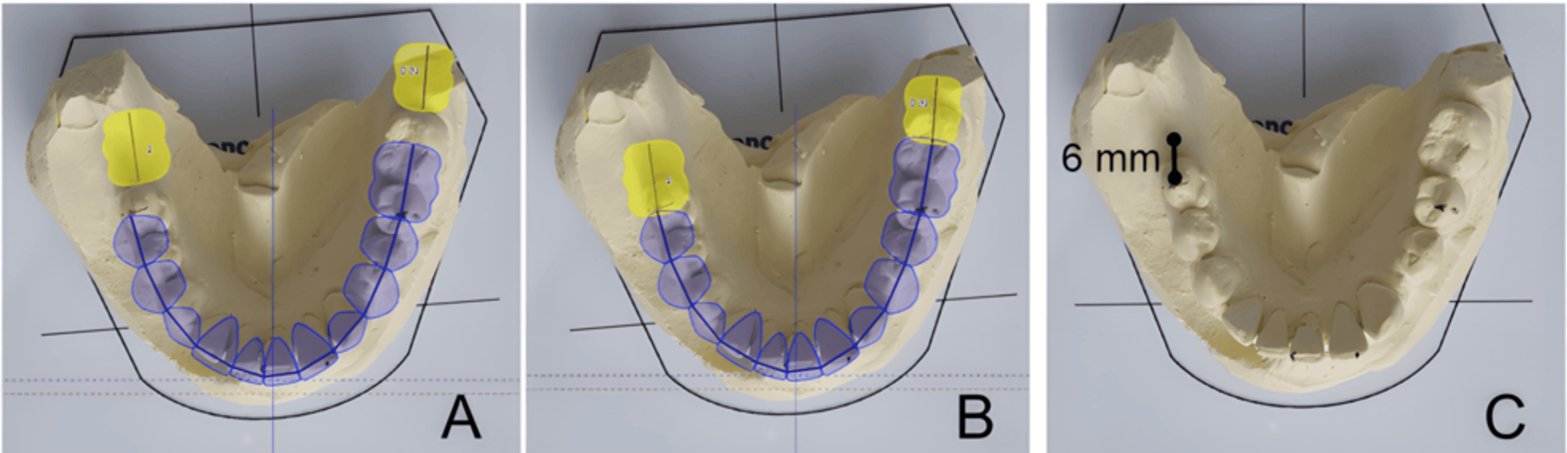
Comparison of implant positioning without vs. with occlusogram-guided planning (A) Implant location determined solely from the current mandibular arch form (yellow outline), demonstrating an improper mesiodistal and buccolingual position that would occur if orthodontic movements were not anticipated. (B) Ideal implant position determined after occlusogram analysis, based on the predicted final arch form and planned mesialization of the posterior segment (yellow outline). (C) Clinical reference used to guide the surgeon: the implant site marked 6 mm apical to the cusp tip of tooth 45 along a line parallel to the occlusal plane, ensuring accurate positioning. This comparison highlights the essential role of occlusogram-guided planning in preventing implant malposition and in communicating precise surgical landmarks for prosthetically driven implant placement Image credit: The image was created using GeoGebra Classic 6 (International GeoGebra Institute, Linz, Austria)

Finally, the occlusogram clearly demonstrated that the implant at site #46 needed to be positioned according to the predicted final arch form rather than the patient's current one (comparison of Figures 6A, 6B). Figure 6A illustrates the improper implant position that would result from placement based on the existing arch configuration, while Figure 6B shows the correct position based on the predicted final arch form, revealing a substantial buccolingual discrepancy of approximately 3-4 mm between these two scenarios. Although the implant appeared buccally positioned relative to the existing arch shape, the occlusogram confirmed that orthodontic movement would subsequently align the dentition with the planned prosthetic emergence profile. Posttreatment clinical assessment verified that the implant-supported restoration exhibited proper buccolingual emergence contour, adequate embrasure spaces, and harmonious integration with the final arch form as predicted by the occlusogram (Figure 6).

Orthodontic treatment

After approximately 12 months, maxillary alignment and midline correction were completed. Following construction of the occlusogram and determination of the correct implant position, a 3.75 mm diameter × 11.5 mm length titanium implant with a moderately rough surface (sandblasted, large-grit, acid-etched) was placed at position #46, with primary stability confirmed by an insertion torque of 35 Ncm. An eight-week osseointegration period was observed before orthodontic loading. Osseointegration was verified through clinical assessment (absence of mobility) and periapical radiographic evaluation (absence of peri-implant radiolucency) before proceeding with orthodontic mechanics.

For the mandibular arch, a rigid lingual arch (0.032-inch stainless steel) was fabricated to connect tooth #36 with the implant at position #46, providing stable anchorage for mesial movement. The lingual arch was secured bilaterally via lingual sheaths (horizontal lingual tubes) soldered to orthodontic bands: one band cemented on tooth #36 and another band adapted to the provisional crown on the implant at position #46. This configuration established the implant as the reactive anchorage unit while orthodontic forces were applied to tooth #36.

**Figure 7:**
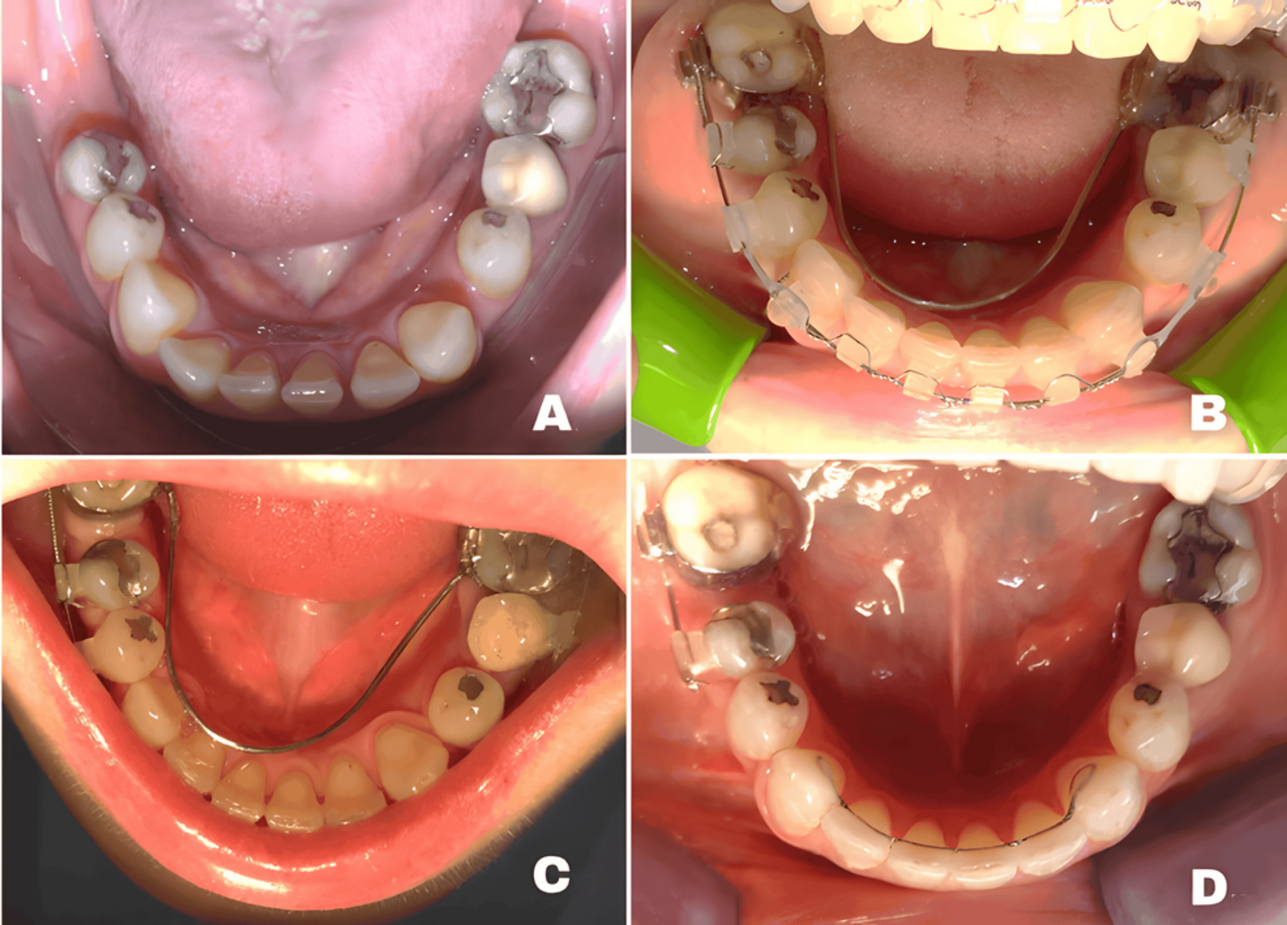
Sequential mandibular orthodontic mechanics using the implant as absolute anchorage (A) Initial mandibular arch before implant placement, showing posterior spacing and need for mesialization. (B) An implant was installed at site 46 and used as an absolute anchorage for mesialization of the contralateral posterior segment, delivered through a preactivated lingual arch configured in Geometry III. (C) Clinical progression after four months of mesialization mechanics, demonstrating a significant reduction of the posterior space. (D) Final mandibular arch form after complete space closure and establishment of the planned arch configuration

The appliance was activated using the truncated V-bend technique, producing a Geometry III-type force system (characterized by predominantly translational movement) appropriate for sagittal movement of the posterior segment. Approximately 200 g of mesial force (verified with a tension gauge during activation) was applied to tooth #36, while the implant served as a stable reactive unit. Implant stability was assessed clinically at each eight-week reactivation (no mobility detected) and radiographically at three-month intervals (maintained osseointegration with no peri-implant bone loss). Periapical radiographs obtained during active treatment confirmed the absence of clinically significant root resorption. The lingual arch was reactivated every eight weeks. After six months, mandibular spaces were successfully eliminated (approximately 8 mm of total space closure through mesial movement of teeth #36, #35, #34, and #33) (Figure 7).

Treatment outcome

**Figure 8:**
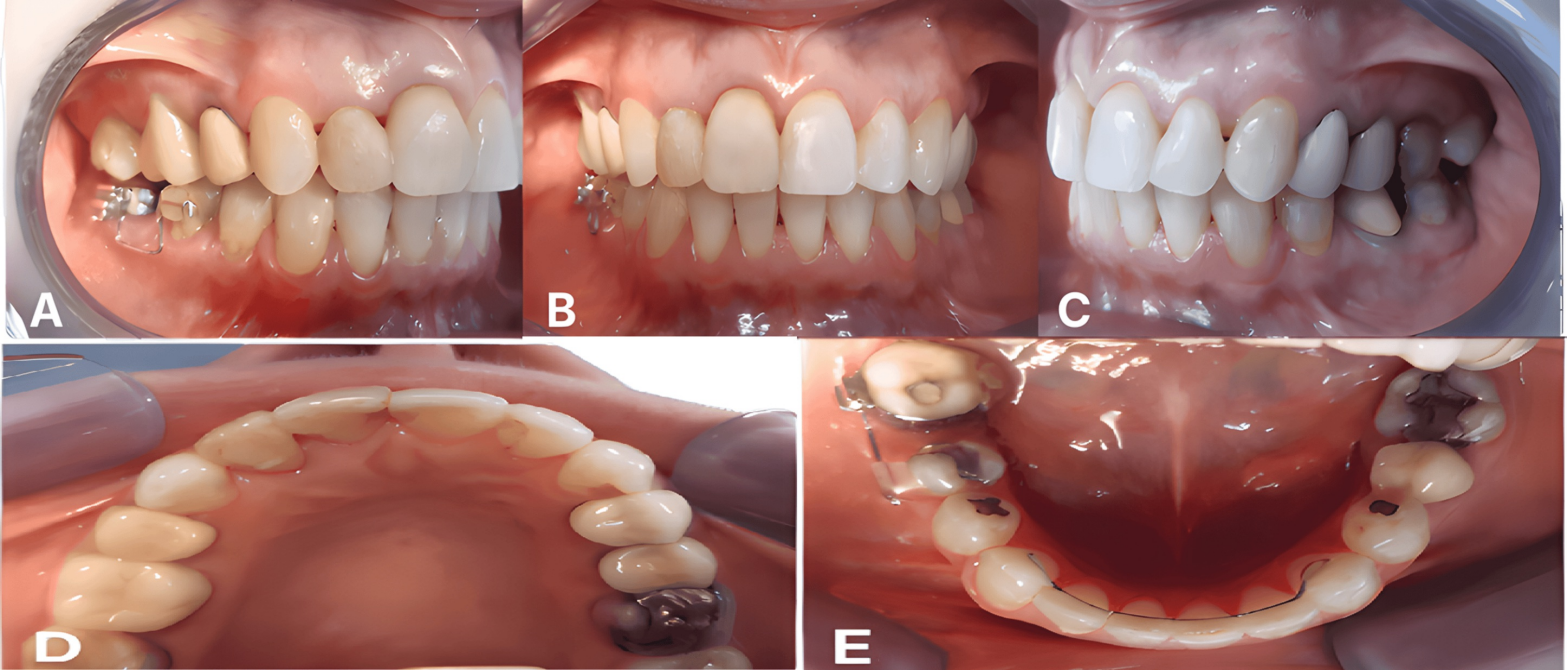
Final treatment outcomes Final intraoral presentation demonstrating achieved treatment outcomes. (A) Right buccal view showing established bilateral Class I canine relationship, proper posterior occlusion with implant-supported crown at position #46, and coordinated intercuspation. (B) Frontal view showing corrected anterior crossbite, proper overbite (2 mm) and overjet (2 mm), maxillary midline centered with facial midline (correcting initial 2 mm deviation), and harmonious dental esthetics. (C) Left buccal view showing maintained Class I canine relationship, closed mandibular spaces, and functional posterior occlusion. (D) Maxillary occlusal view showing aligned arch form and resolved crowding through progressive interproximal reduction. (E) Mandibular occlusal view showing complete closure of 8 mm mandibular spacing through posterior mesialization, implant-supported restoration at position #46 integrated with natural dentition, and harmonious arch form consistent with occlusogram predictions

The implant at site #46 was loaded for orthodontic anchorage eight weeks after placement and served in this capacity for six months during mandibular space closure. Following completion of orthodontic treatment and stabilization (approximately 20 months after initial implant placement), the implant was restored with a definitive crown and functioned harmoniously within the final occlusion. Tooth alignment, midline correction, and closure of mandibular spacing were achieved. Quantitative verification confirmed that treatment outcomes matched occlusogram predictions: 8 mm of mandibular space closure was accomplished as planned, maxillary midline was centered with the facial midline (correcting the initial 2 mm deviation), bilateral Class I canine relationships were established, and the implant-supported restoration exhibited proper buccolingual positioning and occlusal integration as predicted by the occlusogram analysis. The final occlusal relationships were consistent with the occlusogram-based predictions, illustrating the clinical utility of occlusogram-guided planning in this interdisciplinary case (Figures 5, 8).

Figure 6 illustrates the contrast between the ideal implant position determined by the occlusogram vs. the improper position that would have resulted from relying only on the initial arch configuration.

## Discussion

This case demonstrates the clinical application of occlusogram-guided planning in an adult patient requiring orthodontic treatment and implant-supported rehabilitation. The objective was to determine the correct implant location before orthodontic mechanics to enable dual function as anchorage and prosthetic abutment. The occlusogram enabled visualization of planned movements and guided implant placement in harmony with the final occlusal scheme [5], with implant stability confirmed clinically and radiographically throughout the six-month loading period.

In the present case, occlusograms were constructed digitally from standardized occlusal photographs of maxillary and mandibular casts. Once calibrated to a 1:1 scale within GeoGebra software (using the mesiodistal width of the #46 region measured directly on the cast as reference), the occlusal outlines of both arches were traced to represent each tooth's mesiodistal dimension (measured at the greatest mesiodistal convexity between contact points). Traced dimensions were verified against direct caliper measurements on the casts, showing <0.5 mm variation. This allowed simulation of planned tooth movements in the transverse and sagittal planes, reflecting the intended changes in arch form. Superimposition of maxillary and mandibular occlusograms provided a representation of the predicted final occlusal relationships and enabled verification of intercuspation and posterior support [13]. It should be acknowledged that GeoGebra is general-purpose geometric software not specifically designed or validated for dental applications, and that the occlusogram methodology has inherent limitations. The 2D occlusal projection does not capture vertical dimension information, including cusp-tip relationships, buccolingual overlap, or 3D spatial relationships visible in contemporary 3D digital setups. Vertical discrepancies such as supraeruption (evident in tooth #17 in this case) and sagittal curve of Spee alterations are not directly visualized in the occlusogram. In this case, vertical dimension assessment was performed through clinical examination, lateral cephalometric analysis, and visual treatment objective construction, which complemented the 2D occlusogram for comprehensive treatment planning. These limitations should be considered when evaluating the occlusogram as a planning tool, particularly when compared with validated 3D digital workflows that provide complete 3D visualization.

A key advantage was the facilitation of interdisciplinary communication. The occlusogram enabled prediction of final arch configuration and confirmation that the implant could serve dual purposes (anchorage and prosthetic abutment). The simulation identified discrepancies between implant positions based on existing vs. predicted arch forms, using criteria of adequate mesiodistal space (7-8 mm), appropriate buccolingual alignment, root clearance, and proper occlusal contacts. These visualizations were presented during informed consent, demonstrating visually why implant placement required orthodontic planning consideration and facilitating the consent discussion.

The biological and biomechanical capacity of osseointegrated implants to withstand orthodontic loads is supported by experimental and clinical literature [9]. Systematic reviews show that loading protocols and primary stability influence outcomes, underscoring the need for careful force magnitude and timing decisions [4,6]. In this case, the implant provided reliable anchorage for 8 mm of mandibular mesialization over six months, with no mobility detected (clinical and radiographic monitoring) and no unwanted reciprocal movement, consistent with the role of implants as fixed skeletal anchorage [14-16].

Following orthodontic completion, retention included a maxillary Hawley retainer, mandibular fixed canine-to-canine retainer, and a passive 0.032″ segment bonded to #45 and cemented to the provisional crown on #46. This segment prevented periodontal-driven relapse of mesialized teeth during prosthetic fabrication, extending the implant's anchorage role to posttreatment stabilization. The segment was removed when the definitive crown was delivered. Periodontal and peri-implant health were monitored monthly with appropriate hygiene protocols, remaining stable throughout the retention phase.

All planned orthodontic objectives were achieved: anterior crossbite correction, maxillary crowding resolution via IPR, midline correction, mandibular space closure (8 mm mesialization), and coordinated intercuspation with anterior guidance and canine-protected excursions (verified with articulating paper showing bilateral posterior contacts, smooth protrusive guidance, and canine disclusion without balancing contacts). Mild incisor proclination improved profile and lip support, while bilateral posterior support enhanced masticatory function. These outcomes are consistent with prior reports suggesting potential benefits of coordinated implant-orthodontic workflows [4,8,11,12], though comparative studies are needed for definitive evidence.

This case report has several important limitations that should be acknowledged. First, as a single-case report, the findings demonstrate feasibility and clinical application in this specific patient but cannot establish generalizability, treatment time benefits, or comparative effectiveness relative to other planning methodologies. No control group or comparison to 3D digital planning workflows was performed. Second, the occlusogram methodology itself has inherent limitations, as previously discussed: it is a 2D analysis that does not capture vertical dimension, cusp-tip relationships, or buccolingual overlap that are visible in contemporary 3D digital setups. Third, while treatment outcomes were assessed clinically and radiographically, formal patient-reported outcome measures were not collected. Fourth, although three-year follow-up postprosthetic loading has shown stable functional occlusion and maintained peri-implant health radiographically, longer term data would strengthen conclusions regarding treatment stability. Finally, GeoGebra is general-purpose software without formal validation for dental applications, and the reproducibility of this digital occlusogram approach has not been systematically evaluated across different operators or clinical settings. These limitations should be considered when interpreting the clinical utility demonstrated in this case.

In summary, this case illustrates the application of an occlusogram as a visual planning tool that informs both implant positioning and orthodontic sequencing. By anticipating the final occlusion and communicating a shared plan across specialties, this approach may reduce the risk of implant malpositioning, enable effective use of implants as anchorage, and facilitate the pathway to definitive prosthetic rehabilitation.

## Conclusions

This case demonstrates occlusogram-guided planning in an adult patient requiring integrated orthodontic and implant treatment. Using accessible software (GeoGebra), the team simulated final tooth positions to determine implant placement compatible with the predicted occlusion. The implant functioned as anchorage for 8 mm mandibular mesialization and was successfully integrated as a prosthetic abutment, with planned objectives achieved: space closure, Class I canine relationships, midline correction, and functional posterior support. The 2D occlusogram may serve as a low-cost planning adjunct when advanced 3D technology is unavailable, though it has inherent limitations (loss of vertical dimension and lack of 3D spatial visualization) compared with contemporary CBCT-guided and digital workflows. Comparative studies are needed to evaluate accuracy and clinical outcomes relative to validated 3D planning before broader adoption. The feasibility demonstrated here warrants further investigation of this accessible approach.
